# Algorithms to identify COPD in health systems with and without access to ICD coding: a systematic review

**DOI:** 10.1186/s12913-019-4574-3

**Published:** 2019-10-22

**Authors:** Holger Gothe, Sasa Rajsic, Djurdja Vukicevic, Tonio Schoenfelder, Beate Jahn, Sabine Geiger-Gritsch, Diana Brixner, Niki Popper, Gottfried Endel, Uwe Siebert

**Affiliations:** 1 Department of Public Health, Health Services Research and Health Technology Assessment, Institute of Public Health, Medical Decision Making and Health Technology Assessment, UMIT – University for Health Sciences, Medical Informatics and Technology, Eduard Wallnoefer Zentrum 1, A-6060 Hall i.T., Austria; 2 Medical Faculty “Carl Gustav Carus”, Technical University Dresden, Loescherstrasse 18, D-01307 Dresden, Germany; 3 University of Utah, School of Medicine, Salt Lake City, UT 84132 USA; 4 dwh Gmbh, Neustiftgasse 57-59, A-1070, Vienna, Austria; 5 Evidence-Based Medicine and Health Technology Assessment, Main Association of Austrian Social Insurance Institutions, Kundmanngasse 21, A-1031 Vienna, Austria; 6 Division of Health Technology Assessment and Bioinformatics, ONCOTYROL - Center for Personalized Cancer Medicine, Innsbruck, Austria; 7 Center for Health Decision Science, Department of Health Policy and Management, Harvard T.H. Chan School of Public Health, 718 Huntington Ave, Boston, MA 02115 USA; 8 Institute for Technology Assessment and Department of Radiology, Massachusetts General Hospital, Harvard Medical School, 101 Merrimac St, Boston, MA 02114 USA

**Keywords:** COPD, Chronic obstructive pulmonary disease, ICD code, Patient identification, Case finding, Epidemiology, Prevalence, Incidence, Claims data, Routine data, Administrative data, Secondary data

## Abstract

**Background:**

Chronic obstructive pulmonary disease (COPD) causes significant morbidity and mortality worldwide. Estimation of incidence, prevalence and disease burden through routine insurance data is challenging because of under-diagnosis and under-treatment, particularly for early stage disease in health care systems where outpatient International Classification of Diseases (ICD) diagnoses are not collected. This poses the question of which criteria are commonly applied to identify COPD patients in claims datasets in the absence of ICD diagnoses, and which information can be used as a substitute. The aim of this systematic review is to summarize previously reported methodological approaches for the identification of COPD patients through routine data and to compile potential criteria for the identification of COPD patients if ICD codes are not available.

**Methods:**

A systematic literature review was performed in Medline via PubMed and Google Scholar from January 2000 through October 2018, followed by a manual review of the included studies by at least two independent raters. Study characteristics and all identifying criteria used in the studies were systematically extracted from the publications, categorized, and compiled in evidence tables.

**Results:**

In total, the systematic search yielded 151 publications. After title and abstract screening, 38 publications were included into the systematic assessment. In these studies, the most frequently used (22/38) criteria set to identify COPD patients included ICD codes, hospitalization, and ambulatory visits. Only four out of 38 studies used methods other than ICD coding. In a significant proportion of studies, the age range of the target population (33/38) and hospitalization (30/38) were provided. Ambulatory data were included in 24, physician claims in 22, and pharmaceutical data in 18 studies. Only five studies used spirometry, two used surgery and one used oxygen therapy.

**Conclusions:**

A variety of different criteria is used for the identification of COPD from routine data. The most promising criteria set in data environments where ambulatory diagnosis codes are lacking is the consideration of additional illness-related information with special attention to pharmacotherapy data. Further health services research should focus on the application of more systematic internal and/or external validation approaches.

## Introduction

Chronic obstructive pulmonary disease (COPD) is a condition characterized by constriction of the airways, and persistent shortness of breath that interferes with normal breathing. The disease develops over a long period of time and is not fully reversible [1]. COPD is a cause of significant morbidity and mortality. Globally, it is estimated that about three million deaths were caused by the disease in 2015 (i.e., 5% of all deaths globally that year) [2]. The World Health Organization (WHO) reported that COPD was the third cause of mortality worldwide in 2016 [3]. If a COPD diagnosis is made earlier in the progression of the disease, there is a greater potential to reduce further lung damage [4]. For this reason, the identification of COPD patients in early stages of the disease is of great interest for the social health insurance system. Accurate estimates of COPD prevalence are essential for the implementation of strategies for detection and disease management.

The identification of patients suffering from COPD through routine insurance data for a correct measurement and estimation of disease epidemiology and burden of disease turns out to be difficult for various reasons. It is well known that most COPD cases are caused by tobacco consumption over long time periods, but this information, as other life-style-related variables, is generally not available in routine claims datasets. Another reason is underreporting, since there is a very large population of undiagnosed patients with this disease and individuals are undertreated, especially in early stages. In the United Kingdom, for example, there are approximately 835,000 individuals with a diagnosis of COPD, while over 2,200,000 individuals are estimated to be living with undiagnosed COPD [5].

The most commonly practiced approach to filter affected beneficiaries from large datasets (e.g., claims databases) is to apply filter algorithms referring to the International Classification of Diseases (ICD) system, a standard tool in clinical medicine, epidemiology, and health management. Epidemiologists use the ICD system to monitor the incidence and prevalence of diseases and disorders, gaining an insight in the possible health situation of populations and countries. Medical practitioners and clinicians use ICD to identify and to document diseases or other health conditions which can subsequently be archived in health administrative databases and health records. These datasets offer the foundation for the reporting on national mortality and morbidity statistics by WHO Member States. Furthermore, ICD is used for reimbursement purposes and for decision-making regarding resource allocation by many countries [6].

Identifying COPD patients in the absence of ICD codes in a large dataset is challenging, as it requires the combination of other suitable identifiers, which may be included in the data, such as pharmacy based health plans (PBMs) in the US, South Africa, or in Europe. For example, in the Austrian outpatient system the ICD code is not available in routine data, and therefore identifying COPD patients via medical claims is even more difficult. Thus, the Main Association of Austrian Social Insurance Institutions (“Hauptverband der österreichischen Sozialversicherungsträger”) likely uses advanced mathematical methods to identify COPD patients with available routine data.

## Aim

The goals of this study are to summarize previously reported methodological approaches to identify COPD patients through routine data, and to compile potential surrogate criteria for the identification of COPD patients when ICD codes are not available.

## Methods

### Information sources

A systematic literature review was performed in Medline via PubMed and Google Scholar, followed by a manual review of the included studies. Medline via the PubMed interface was used to conduct separate literature searches in the English or German language from January 2000 through October 2018. The systematic literature search was performed with the following algorithm: *(“epidemiology” OR “prevalence” OR “incidence”) AND (“COPD” OR “chronic obstructive pulmonary disease”) AND (“claims data” OR “routine data” OR “administrative data”).*

To ensure maximum completeness of the search, we performed a reference list search of the included studies for additional relevant citations via Google Scholar. We did not search the Internet to assess available grey literature. Each included study was summarized narratively and presented in evidence tables with regard to the study aim, datasets used and the identification criteria for COPD patients. In studies where sensitivity analysis of the algorithm regarding the correct identification of COPD was performed, these results were reported.

### Literature screening process, inclusion and exclusion criteria

The title and abstract screening was conducted by three authors (SR, DV, SG), based on predetermined selection criteria (see below). In case of incongruence, a fourth assessor (HG) made a final decision on the eligibility of a publication. The full-text articles of selected studies were further reviewed by at least two authors and included if they met all inclusion criteria.

Publications were included if authors agreed on all of the four following selection criteria: (1) at least one secondary data set was used in the study, (2) COPD was identified in a population with suspicion of being diseased, (3) available information from a routine dataset was used, and (4) identification criteria for COPD were clearly explained.

Studies were excluded if they primarily reported on diseases other than COPD or if the addressed intervention (e.g., thoracic surgery) was irrelevant. We excluded all studies enrolling pre-diagnosed COPD patients, for whom there was no need to show any identification algorithms, as these studies would not help answer our research question. Similarly, publications were excluded if the COPD identification algorithms were not revealed in the text, or if they consisted only of a study protocol.

### Data extraction and reporting

We extracted descriptors of the studies and related publications as well as characteristics commonly used for the description of COPD populations. We pre-defined the following data to be extracted from the publications: author(s), year of publication, publication title, country of conduct of the study, dataset(s) used, age range, ICD codes, hospitalization data, ambulatory visit data, physician claims data, ambulatory pharmacotherapy, spirometry data, oxygen therapy data, COPD-related surgical procedure, and algorithm of COPD diagnosis. Data extraction was performed by one assessor and validated by a second assessor.

Existing risk of bias tools such as the Cochrane risk of bias tool for randomized controlled trials [7], the Newcastle-Ottawa Quality Assessment Form for Cohort Studies [8], and the ROBINS-I tool for assessing risk of bias in non-randomized studies of interventions [9] are not applicable to studies using administrative data analyses. Until now, no well-accepted specific tools for these kinds of studies are available; we therefore used the method of algorithm validation within our studies to judge the risk of bias. Specifically, the risk of bias was appraised by classifying the studies into two risk groups: (1) “low risk of bias” if the used algorithm was validated against a reference standard with sensitivity and specificity greater than 70% and (2) “high risk of bias” if the algorithm was not validated or sensitivity was lower than 70%.

The review was conducted according to PRISMA - Preferred Reporting Items for Systematic Reviews and Meta-Analyses [10]. Results are reported as standardized narrative summaries of the included studies and as an evidence table for the identification criteria utilized in the included studies. The different instruments, methods, and algorithms to identify COPD patients, the databases used and related challenges are discussed in detail.

## Results

### Included studies

The search yielded 151 hits in Medline via PubMed, with the last update in October 2018. After title and abstract screening, 104 papers were excluded for the following reasons: 52 studies addressed a disease other than COPD, in 31 studies patients were identified without disclosing the algorithm or because the patients’ COPD status was known at the beginning of the study, 17 studies described an irrelevant intervention or condition (e. g., COPD not in the focus of the analysis) and four studies were protocols only. Search via Google Scholar did not yield any citations beyond the Medline search, while the hand search of the included studies reference lists revealed one more study, which was included (Mapel et al. 2006 [11]).

**Fig. 1:**
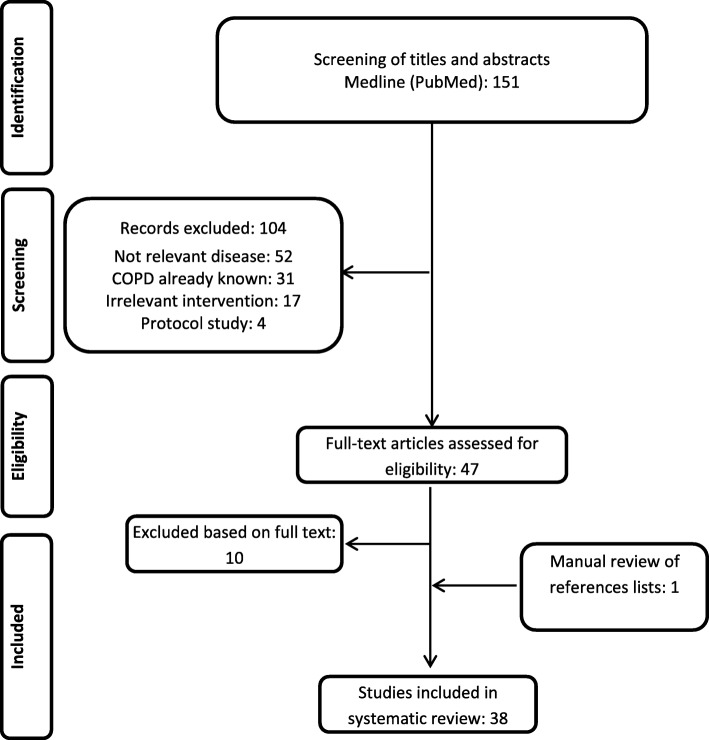
PRISMA flowchart reporting the inclusion/exclusion of publications into/from the review

Forty-seven papers were included for full-text screening (see Fig. 1), 10 of them were excluded due to the following reasons: Two publications (Chu et al. 2010 [12], Schneider et al. 2009 [13]) were excluded, because they focused on general aspects of COPD or chronic diseases. Thus, both publications do not specify which algorithms were used for the identification of COPD patients from the datasets. Eight studies were excluded, because they used ICD codes only (Albrecht et al. 2016 [14]; Fortin et al. 2017 [15]; Schwarzkopf et al. 2016 [16]), or because they only reported the study protocol (Josephs et al. 2017 [17]), or because they did not differentiate between asthma and COPD (Marrie et al. 2016 [18]; Oelsner et al. 2016 [19]). One publication was excluded, because it duplicated another publication (Vozoris et al. 2016 [20]), and one study was excluded, because it investigated a different disease (Pollmanns et al. 2018 [21]). Finally, 38 studies were included in the review as one study was identified by hand search.

Included studies predominantly reflect the situation of North American countries: United States (*n* = 17) and Canada (*n* = 17). Four studies reported on the COPD identification process in Europe: United Kingdom (*n* = 1); Italy (*n* = 2) and France (*n* = 1) (Tables 1 and 2).

**Table 1:** Identification criteria utilized in the published studies. Part A: Studies with low risk of bias (in chronological order)

Authors, publication year, country	Dataset used	Study population / COPD population	Age limitation	ICD codes	Hospitalization	Ambulatory visit	Physician claims	(ambulatory) Pharmacotherapy	Spirometry	Oxygen therapy	COPD-related surgical procedure	Algorithm	Risk of bias (low or high)
Gershon et al. (2009) Canada [22]	- Ontario Health Insurance Plan - Canadian Institute of Health Information discharge abstract Db	SP: 442 COPD-P: 113	X		X	X						Years > 35. At least one ambulatory claim and/or at least one COPD hospitalization	low (validated algorithm: sensitivity 85%, specificity 78.4%. Reference standard: expert opinion)
Gershon et al. (2010) Canada [23]	- Ontario Health Insurance Plan Db - Canadian Institute for Health Information Db records - Ontario Registered Persons Db	*1996:* SP: 5,548,341 COPD-P: 430,000 *2002:* SP: 6,444,492 COPD-P: 603,770 *2007:* SP: 7,082,086 COPD-P: 708,743	X	X	X	X	X					Years > 35. At least one COPD physician billing claims and /or at least one COPD hospital discharges (ICD code 491, 492, 496 or J41-J44).	low (validated algorithm: sensitivity of 85.0% and a specificity of 78.4% - Gershon et al. 2009)
Cooke et al. (2011) United States [24]	- Two departments of Veterans Affairs inpatient and outpatient Db	SP: 9,573 COPD-P: 4,564	X	X	X	X		X	X			Years ≥40 1. FEV1/FVC < 0.70 2. FEV1/FVC < lower limits than normal.	low (Algorithm: sensitivity 72%, specificity 74%. Reference standard: spirometry)
Gershon et al. (2011) Canada [25]	- Registered Persons Db - Canadian Institute of Health Information Discharge Abstract Db - Ontario Health Insurance Plan Physician claims data	SP: 13,022,536 COPD-P: 579,466	X	X	X	X						Years > 35. ICD-9491, 492, 496; ICD-10 J41-J44. One hospitalization or One ambulatory care visit (general definition) / three or more ambulatory care visits in a 2 year period (second, specific COPD definition).	low (validated algorithm: sensitivity of 85.0% and a specificity of 78.4% - Gershon et al. 2009)
Austin et al. (2012) Canada [26]	- The Ontario Chronic Obstructive Pulmonary Disease Db - The Registered Persons Db - The Canadian Institute for Health Information, Discharge Abstract Db - The Ontario Health Insurance Plan physician billing Db - The Ontario Mental Health Reporting System	COPD-P: 216,735 incident COPD-P: 638,926 prevalent	X	X	X		X					Years > 35 1. At least one physician billing claims or 2. At least one COPD hospital discharge: ICD-9 codes 491, 492, or 496; ICD-10 codes: J41, J42, J43 or J44.	low (Algorithm: sensitivity 85.0%, specificity 78.4%. Reference standard: expert opinion-based on Gershon et al. 2009)
Gershon et al. (2013) Canada [27]	- Registered Persons Db - Canadian Institute of Health Information Discharge Abstract - National Ambulatory Care Reporting System Db - Ontario Health Insurance Plan Physician Claims Db - Ontario Home Care Db - Ontario Drug Benefits Db	SP: 7,246,982 COPD-P: 853,438	X		X	X	X					Physician-diagnosed COPD: 1. Years > 35 and 2. Having one hospitalization related to the COPD and/or 3. One ambulatory care claim related to the COPD.	low (Algorithm: sensitivity 85.0%, specificity 78.4%. Reference standard: expert opinion-based on Gershon et al. 2009)
Gershon et al. (2014) Canada [28]	- The Registered Persons Db - The Canadian Institute of Health Information Discharge Abstract Db - The Ontario Health Insurance Plan Physician Claims Db	SP: 13,000,000 COPD-P: 807,046	X	X	X		X					Years ≥35. 1. At least one COPD physician billing claims and/or 2. At least one COPD hospital discharge as per the following codes: 491, 492, 496 ICD-9 or J41, J42, J43, J44 ICD-10	low (Algorithm sensitivity 85%, specificity 78% when compared with clinical evaluation)
Gershon et al. (2015) Canada [29]	- The Registered Persons Db - The Canadian Institute of Health Information Discharge Abstract - The Ontario Health Insurance Plan Physician Claims Db - Ontario Registrar General Death Db	SP: 7,626,745 COPD-P: 836,139	X	X	X	X	X					Years > 35 COPD: ICD-9 and ICD-10 1. At least one COPD physician billing claims and/or 2. At least one COPD hospital discharge.	low Algorithm: sensitivity 85.0%, specificity 78.4%. Reference standard: expert opinion (based on Gershon et al. 2009).
Crighton et al. (2015) Canada [30]	- The Registered Persons Db - The Canadian Institute of Health Information Discharge Abstract Db - The Ontario Health Insurance Plan Physician Claims Db - National Ambulatory Care Reporting System Db	SP: NA COPD-P: 722,494	X	X	X	X	X					Years ≥35. 1. One or more COPD hospitalizations and/or 2. One ambulatory care claim (ICD-9: 491, 492, 496 or ICD-10: J41, J42, J43, J44)	low Algorithm: sensitivity 85.0%, specificity of 78.4%. Reference standard: physician clinical evaluation
Doucet et al. (2016) Canada [31]	- linked health administrative data: (1) the health insurance registry of the R’egie de l’assurance maladie du Qu’ebec (RAMQ), (2) fee-for-service data (physician billing), (3) hospital discharge (4) drug data for the 65 years and older, (5) mortality data.	SP: NA COPD-P: 444,709	X	X	X		X					1. One or more visits to a physician 2. One hospitalization with a COPD diagnosis 3. Years ≥35 ICD-9 codes 491–492 and 496 or ICD-10-CA J41–44.	low (validation against clinical reference standard: sensitivity of 85% (95% CI: 77.0 to 91.0%) and a specificity of 78.4% (95%CI: 73.6 to 82.7%)
Romanelli et al. (2016) Italy [32]	- Hospital discharge register (HDR) - The cause-specific mortality register (CMR) - Clinical and spirometric data from clinical (hospital or outpatient) charts at the Institute of Clinical Physiology (ICP) of the National Research Council (NRC)	SP: NA COPD-P: 2,544	X	X	X	X			X			years ≥40 1. Hospital discharge with a primary or secondary COPD diagnosis (ICD-9: 490, 491, 492, 494, 496) or 2. Received a diagnosis of COPD in hospital or outpatient charts or 3. FEV1/FVC < 0.70 at spirometry or 4. COPD as a cause of death.	low (validation from clinical and spirometric data)
Gershon et al. (2017) Canada [33]	- 4 government health administrative databases: (1) The Registered Persons Database (2) The Ontario Health Insurance Plan Physician Claims database (3) The Canadian Institute of Health Information Discharge Abstract database (4) the National Ambulatory Care Reporting System Data were linked using unique encoded identifiers	SP: NA COPD-P: 874,336	X	X	X	X						Years ≥35. 1. One or more COPD ambulatory care visits and/or 2. One or more COPD hospitalizations COPD ICD-10 codes J41–J44	low (validation against clinical reference standard was 85.0% sensitivity and 78.4% specificity)
Lee et al. (2017) Canada [34]	data from the Electronic Medical Record Administrative data Linked Database (EMRALD®)	SP: 5,889 COPD-P: 364	X	X			X	X				years ≥35 several electronic medical record algorithms; the one with best validation results included: 1. Three or more physician billing codes for COPD per year; 2. Documentation in the cumulative patient profile (CPP); 3. Tiotropium prescription; or ipratropium (or its formulations) prescription and 4. A COPD billing code	low (validation against an abstracted patient chart reference standard: sensitivity of 76.9% (95% CI:72.2–81.2), specificity of 99.7% (99.5–99.8)
McGuire et al. (2017) Canada [35]	- Ministry of Health of British Columbia administrative databases on provincially funded health services. PharmaNet data on all medications - Data on deaths (from death certificates)	SP: 50,021 COPD-P: 594		X	X		X					ICD-9 codes 491, 492, 493.2, 496, or ICD-10 codes J43 or J44 in hospital/outpatient physician visit data. Primary outcome: first COPD hospitalization	low (used an algorithm which was validated against a clinical reference standard by Gershon et al. 2009)
Westney et al. (2017) United States [36]	- Medicaid Analytic eXtract (MAX) file from Centers for Medicare and Medicaid Services	SP: NA COPD-P: 291,978	X	X	X	X						Years 18–64 1. ICD −9 codes 491.0, 491.1, 491.2, 491.8, 492.xx, 493.2, 494.xx, 496.xx and 2. One or more inpatient billed claims from the inpatient file or at least two outpatient billed claims	low (used a validated algorithm from Gershon et al. 2009)

The next-to-last column on the right gives the identification criteria based on the statements contained in the publication

*SP* Study population, *COPD* COPD-P population, *Db* Database, *NA* Not available; see also list of abbreviations

**Table 2:** Identification criteria utilized in the published studies. Part B: Studies with high risk of bias (*n* = 23, in chronological order)

Authors, publication year, country	Dataset used	Study population / COPD population	Age limitation	ICD codes	Hospitalization	Ambulatory visit	Physician claims	(ambulatory) Pharmacotherapy	Spirometry	Oxygen therapy	COPD-related surgical procedure	Algorithm	Risk of bias (low or high)
Hansell et al. (2003) United Kingdom [37]	- Office for National Statistics - Hospital Episode Statistics - General Practice Research Db - Health Survey for England 1995	SP: NA COPD-P: NA		X	X	X	X	X				COPD: ICD-9: 490–492, 494–496. COPD symptoms: cough or phlegm for at least 3 months during the winter	high (no validation of algorithm)
Wilchesky et al. (2004) Canada [38]	- Quebec universal medical insurance register - Medical services claims data	SP: NA COPD-P: 14,980	X	X			X					COPD: 490–490.9, 494–494.9, 496–496.9 Diagnostic criteria: 1. Years ≥66. 2. Two visits or more to the MOXXI physicians in the year.	high (validation against diagnostic codes from medical charts: low sensitivity 45.97 [43.9,48.0] but higher specificity 88.42 [87.9,89.0]
Lacasse et al. (2005) Canada [39]	- Quebec universal medical insurance register	SP: 2,487,605 COPD-P: 176,313	X	X	X	X	X	X				Years ≥65. COPD: IDC-9: 491, 492 and 496. Possible COPD: Years ≥65 or older, always registered as COPD (never as asthmatics), appeared three times in the database and who field prescription for ipratropium bromide or beta2-agonist Probable COPD: all above plus internist or pulmonologist COPD diagnosis.	high (no validation against reference standard; sensitivity and specificity were not determined)
Mapel et al. (2006) United States [11]	- Lovelace Health Plan, a health maintenance organization serving New Mexico	SP: 41,428 COPD-P: 2129	X	X	X	X		X				Years ≥40. Any patient with one or more claims records with COPD diagnosis (491, 492, 496). Excluded: ICD-9140–208, 494, 405, 500–519, 173, 174, 185.	high (algorithm: low sensitivity 60.5%, specificity 82.1%. Reference standard: COPD diagnosis abstracted from medical record, based on ICD codes.)
Akazawa et al. (2008) United States [40]	- United Healthcare claims data (medical and pharmaceutical)	SP: 81,322 COPD-P: 28,968	X	X	X		X	X				Years ≥40. 1. Inpatient hospital or emergency room bill with a diagnosis with ICD code: 491, 492, 496; or 2. Physician claims with a COPD diagnosis and a second COPD-related medical claim with a separate date; or 3. Physician claims with a COPD diagnosis with pharmacy claim for certain medication.	high (no validation of algorithm)
Heins-Nesvold et al. (2008) United States [41]	- Managed care administrative Db (Medical and pharmacy administrative claims data, Midwest health) - Mailed survey to COPD patients in Minnesota	SP: NA COPD-P: 7782	X	X	X	X	X	X	X	X		Cases were identified through enrolment, pharmacy and medical files. 2. Years ≥40 3. At least one claim with a COPD diagnosis 4. Claim with the COPD associated diagnosis initiated from a “medical” place of service.	high (no validation of algorithm)
Mapel et al. (2010) United States [42]	- Lovelace Health Plan	SP: 10,904 COPD-P: 2707	X	X	X	X		X				Years ≥40. COPD: ICD-9 code 491, 492, 496. Continuously enrolled for 2 years prior to index date, one inpatient or two outpatient claims with COPD-related ICD code and National drug COPD-related code.	high (algorithm: specificity 70.5%, but low sensitivity 60.6%. Reference standard: COPD diagnosis in a medical record)
Dalal et al. (2011) United States [43]	- IMS Lifelink Db	SP: NA COPD-P: 9188	X					X				Years ≥40 PD maintenance medication pharmacy claim, specially inhaled corticosteroids, long-acting beta-agonists, anticholinergics, and/or fixed-dose combination regimes.	high (no validated algorithm)
Mapel et al. (2011) United States [44]	- US managed care administrative claims data (multiple health plans)	Commercial insurance: SP: 7,671,018 COPD-P: 42,565 Medicare insurance: SP: 115,652 COPD-P: 8507	X	X	X	X	X				X	1. Years ≥40; one inpatient COPD hospitalization or emergency department visit (ICD 491, 492, 496); or 2. Years ≥40; two professional COPD claims (different service dates); or 3. Years ≥40; surgical procedure related to the COPD (lung volume reduction).	high (no validated algorithm)
Dalal et al. (2012) United States [45]	- Ingenix Impact National Benchmark Db	SP: NA COPD-P: 1936	X	X				X				1. Years ≥40 2. Continuously enrolled 3. Received maintenance therapy: anticholinergic or fluticasone propionate/salmeterol combination within 1 month after an index event COPD IDC-9 code: 491, 492 and 496	high (no validated algorithm)
Make et al. (2012) United States [46]	- PharMetrics Db including 12,4 million covered lives	Commercial insurance: SP: 7.671,018 COPD-P: 42,565 Medicare: SP: 115,652 COPD-P: 8507	X	X	X	X	X				X	Years ≥40. Any of the following: 1. One inpatient COPD-related hospitalization or one emergency department visit (ICD-9: 491, 492, 496) 2. Two professional COPD claims (different service dates) 3. Surgical procedure related to the COPD listed on a facility or professional claim. At least one filled prescription for drug during the study period was enough to consider the patient as to be taking medication. Maintenance COPD pharmacotherapy: LABA, SAAC, LAAC, theophylline and inhaled corticosteroids. SABA was considered symptomatic medications.	high (no validated algorithm)
Gini et al. (2013) Italy [47]	- Hospital discharge records - Drug dispensing records - Disease-specific exemption from co-payment to health care - Inhabitant Registry	SP: 11,656 COPD-P: NA		X	X	X		X				Identification of COPD patients was performed with use of: Hospital discharge records (ICD codes: 490–492; 494, 496); drug dispensation records (ATC code); general physician data (ICD code: 490–492, 494, 496).	high (no validation of algorithm)
Macaulay et al. (2013) United States [48]	- Geisinger Health System (GHS)	SP: NA COPD-P: 2028		X	X	X	X	X	X			COPD ICD-9 codes: 491, 492 or 496 . Results from at least one spirometer test Reference standard: COPD diagnosis (using ICD-9 codes) and electronic health record results from at least one spirometry test.	high Validation of COPD severity Code: low sensitivity, high specificity only for severe/very severe category)
Yawn et al. (2013) United States [49]	MarketScan® Db: 1. Commercial Claims and Encounters 2. CMS Supplemental and Coordination of Benefits	SP: 1,669,546 COPD-P: 135,445	X	X	X	X		X				Years ≥45. COPD: ICD - 9 codes: 491, 492 or 496. 1. Admissions or emergency department visits or at least two COPD-related office visits with different service dates. A continuous enrolment of patients was required for the period of 1 year before the COPD diagnosis and at least 2 months after the COPD diagnosis date. Excluded: with a history of ICS use or pneumonia in the 1 year baseline period; asthma, cystic fibrosis and lung cancer.	high (no validated algorithm)
Dore et al. (2014) United States [50]	- Normative Health Information Db (UnitedHealth Care)	SP: NA COPD-P: 225,079 LABA Users	X	X				X				Years > 20. COPD: ICD-9491.2, 492.8, 496. - Top 3 variables predictive for COPD confirmation: 65 or older, inhaled anticholinergic drug and radiologic examination of the chest - Claim for COPD Only: medications, prescriber specialty, diagnoses, spirometer procedure.	high (Validation of algorithm against medical records – low sensitivity)
Erdem (2014) United States [51]	- Chronic Conditions Public Use Files (Centers for Medicare and Medicaid Services)	SP: NA COPD-P: NA		X	X	X						COPD: ICD-9 code, CPT-4 code or the HCPCS code.	high (no validated algorithm)
Vozoris et al. (2014) Canada [52]	- Ontario Health Insurance Plan claims Db - Canadian Institute for Health Information Discharge Abstract Db - Ontario Mental Health Reporting System - National Ambulatory Care Reporting System Db - Same-Day Surgery Db - Registered Persons Db - Ontario Drug Benefit claims Db	SP: NA COPD-P: 177,355	X	X	X	X	X					Years > 66. COPD: At least three ambulatory claims for COPD within 2 years, or at least one COPD hospitalization	high Algorithm: specificity 95.4%, low sensitivity 57.5%
Aldrich et al. (2015) United States [53]	- Center for Medicare and Medicaid Services encounter	SP: 26,927 COPD-P: 20,945	X	X	X	X	X					Years 40–79. COPD diagnoses defined by using two previously published algorithms (Stein et al. 2012, Mapel et al. 2011). 1. Mapel: one or more COPD hospitalization or emergency department visit (ICD-9491, 492, 496) or at least two professional claims (different service dates) 2. Alternatively, a primary discharge COPD diagnosis (ICD-9491.21) throughout the same period of time following algorithm four defined by Stein et al.	high Validity: low sensitivity 62% and positive predictive value of 80% for identified COPD. Reference standard: COPD diagnosis in reviewed medical record
Vozoris et al. (2015) Canada [54]	- Ontario Drug Benefit Db - Ontario Health Insurance Plan Db - Canadian Institute for Health Information Discharge Abstract Db - National Ambulatory Care Reporting System Db - Ontario Mental Health Reporting System - Same-Day Surgery Db - Ontario Cancer Registry - Database of Ontario adults with physician-diagnosed congestive heart failure - Registered Persons Db	Community dwelling: 107,109 Long-term care resident: 16,207	X	X	X	X	X					Years ≥66. COPD diagnosis algorithm used three or more COPD ambulatory claims within a period of 2 years or at least one COPD hospitalization (specificity 95.4%, sensitivity 57.5%) 1. Three or more ambulatory claims for COPD within 2 year period or 2. One or more hospitalizations for COPD 3. Medication records.	high Algorithm: specificity 95.4% [95% CI 92.6–97.4%]; low sensitivity 57.5% [95% CI 47.9–66.8%])
Laforest et al. (2016) France [55]	- the Permanent Sample of Health Insurance Beneficiaries (EGB): a 1/97th random sample of the French National Claims Data beneficiaries (SNIIRAM) with individual linkage between primary (ambulatory) and secondary (hospital) care	SP: 4237 COPD-P: 4237	X	X	X			X				years ≥45 1. COPD related hospitalization (ICD-10 codes J41, J42, J44 and J96.1.. The J96.0 was accepted as primary diagnosis only if J43 or J44 were present) 2. Long-term disease status for COPD (ICD-10 codes J41, J42, J44 and J96.1) 3. Bronchodilator drugs (LABA, SABA, LAMA, SAMA, xanthines, and SAMA/SABA fixed combinations.	high (no validation of algorithm against clinical reference standard)
Price et al. (2016) United States [56]	Clinformatics™ Data Mart retrospective claims database: - include medical claims (primary and secondary care), - pharmacy claims and - laboratory test results	SP: 93,980 COPD-P: 6687	X	X			X	X				years 4–64 1. Diagnosis of COPD and/or 2. Exercise induced bronchoconstriction recorded at any time and 3. At least one prescription for albuterol	high (no validation of algorithm against clinical reference standard)
Raymakers et al. (2017) Canada [57]	- PharmaNet prescription data - Discharge Abstract Database - British Columbia Vital Statistics Deaths - The regional health authority and census neighborhood income data - Physician billing data from the provincially administered universal insurance program	SP: 39,678 COPD-P: 41,602	X					X				years ≥50 1. Three or more prescriptions (anticholinergic or a short-acting beta agonist) in a 12-month period Index date: the date of receipt of the first prescription	high (no validation of algorithm against clinical reference standard)
Turner et al. (2018) United States [58]	- HealthCore Integrated Research Database - Medical records	SP: 2,219,034 COPD-P: 17,156	X	X			X	X	X			years ≥40 1. ≥2 COPD diagnoses (ICD-9 CM codes 491, 492, 496), 2. ≥2 COPD-related procedures, 3. ≥3 Generic Product Identifier (COPD medication prescription fills) and 4. ≥2 Current Procedural Terminology codes for spirometry tests	high medical record review: COPD confirmation by persistent airflow obstruction FEV1/FVC < 0.70 at symptom baseline; but missing data constrained COPD identification

The next-to-last column on the right gives the identification criteria based on the statements contained in the publication

*SP* Study population, *COPD* COPD-P population, *Db* Database, *NA* Not available; see also list of abbreviations

This review covers a publication period of 16 years as the first study was published in 2003 (Hansell et al.). In the first 8 years (2003–2010), nine articles were published, while in the next 8 years (2011–2018), 29 studies (76.3%) were published.

The classification into high and low risk of bias according to the performed validation of algorithm, resulted in 15 studies with “low risk of bias” due to a validated algorithm with a sensitivity and specificity higher than 70%, whereas 23 studies either did not use a validated algorithm (*n* = 14) or the validation of their algorithm revealed a sensitivity lower than 70% (*n* = 8) or missing data limited validation (*n* = 1) (Tables 1 and 2).

### Identification criteria used in the included publications

**Fig. 2:**
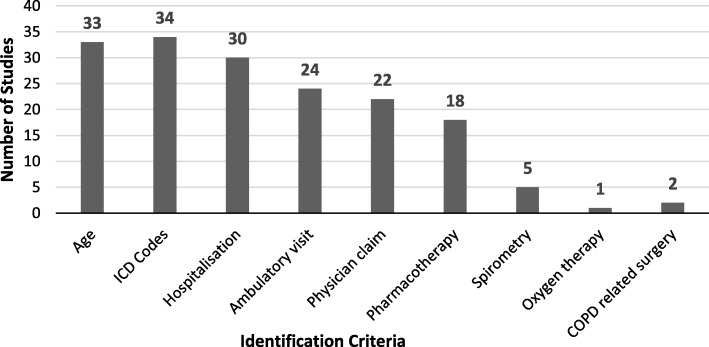
Criteria used for identification of chronic obstructive pulmonary disease across included studies

In this review, ICD coding was the most common variable to identify COPD patients. In 34 of 38 studies ICD-9 (codes from 490 to 496) or ICD-10 (codes from J41 to J44) coding were used as one part of the identification process, while four studies used other methods. In a significant proportion of studies hospitalization data (30 of 38) and the age range of the target population (33 of 38) were provided. Gershon et al. (2009) [22] and Gershon et al. (2013) [27] used age limitation, and one or more hospitalizations or ambulatory claim as indicators for COPD; while Dalal et al. (2011) [43] used age range and pharmacotherapy claim. Ambulatory data were included in 24 studies, physician claims in 22 studies, and 18 studies stated some kind of pharmaceutical data. Only five studies used spirometry data as part of the identification process and one study used information about home oxygen use (Fig. 2. Criteria used for identification of COPD in the studies). Different combinations of these indicators were used in order to identify COPD patients in assessed studies, showed in Tables 1 and 2. Studies that report on the validity of using a specific approach or algorithm to identify COPD patients carry a corresponding indication in the last column of Tables 1 and 2.

The most common combination of identification criteria (22 out of 38 studies) included ICD codes, hospitalization, and ambulatory visits. The next most common combination (12 out of 38 studies) was adding physician claims to the former three criteria. The next adjoining indicator added to one of these two combinations was a prescription claim.

### Studies using identification criteria other than ICD codes

Gershon et al. (2009) [22] and Gershon et al. (2013) [27] used other methods than ICD coding. Both studies published by Gershon et al. used an age limitation and one or more claims for hospitalization or ambulatory care as indicators for COPD. Dalal et al. (2011) [43] and Raymakers et al. (2017) [57] used age range and pharmacotherapy claims.

Gershon et al. (2009) [22] conducted a validation study for population-based administrative COPD definitions. For this validation, two Canadian data sources were used. The first database was the Ontario Health Insurance Plan, which contains hospital and outpatient claims for populations in Ontario (including information on laboratory tests, physicians visit, and diagnostic imaging). As part of a physician claims, the ICD code was provided (ICD-9 codes: 491–492, 496 and ICD-10 codes: J41, J43-J44). The second database contained administrative and clinical data for each hospital visit, coded with ICD-10 (the Canadian Institute of Health Information discharge abstract database). Reference standard diagnoses of each patient were associated with their health administrative record using the insurance number. Furthermore, using the concept of diagnostic test evaluation, reference standard diagnoses were compared to the predefined COPD definitions and analyzed.

In total, 442 medical charts were used in this study, of which 113 medical charts belonged to COPD patients. An expert panel of two pulmonologists examined patients` charts and COPD has reliably been diagnosed by pulmonary function tests. The most sensitive health administrative COPD definition (sensitivity 85.0%, specificity 78.4%) referring to expert opinion and clinical diagnosis included one or more ambulatory claims and/or one or more COPD hospitalizations.

A highly specific COPD definition, with sensitivity of 57.5% and specificity of 95.4%, included the following criteria:

- Patients aged ≥35 years with one or more hospitalizations, or three or more ambulatory care visits for COPD within a two-year time period (definition 1). When the time period was increased to 3 years, specificity remained the same (95.4%), but sensitivity increased to 59.3% (definition 2). The algorithm with the most sensitive definition of COPD (sensitivity of 85.0% and specificity of 78.4%) was one or more hospitalizations, or one or more ambulatory care visits for COPD within an unspecified time period (definition 3).
- ICD-9 codes: 491, 492, 496; ICD-10 codes: J41-J44 [22].

In their later published papers, Gershon and colleagues used definition 3 with the most sensitive definition of COPD as described above (sensitivity of 85.0% and specificity of 78.4%) [23, 27–29]. In one study they also used the highly specific COPD definition 1 (one hospitalization or one or more ambulatory care claim for COPD in adults aged ≥35 years) with sensitivity of 57.5% and specificity of 95.4% [25]. Gershon’s definition 1 with 95.4% specificity (95%CI 92.6–97.4%) and 57.5% sensitivity has also been used by other authors analyzing administrative claims data [52, 54].

Dalal et al. (2011) [43] performed a study to estimate the impact of cardiovascular disease on costs and healthcare utilization in a COPD population in the United States. The data was obtained from the IMS Lifelink claims database, including pharmacy and medical data (demographic data, prescription records, outpatient and inpatient procedures and diagnoses). In total, 9188 patients were analyzed.

Raymakers et al. (2017) [57] investigated the association of statins use with all-cause mortality in patients with COPD. The authors used various administrative and health databases. COPD patients were identified as 50 years old or older, with three or more medication prescriptions (anticholinergic or a short-acting beta agonist) in a one-year period. In total, 39,678 patients were analyzed.

### Studies using identification criteria including ICD codes

In 34 of 38 studies, ICD-9 or ICD-10 codes were used to identify COPD patients. The characteristics of these studies are displayed in Tables 1 and 2. Thirteen of these studies report on the validity of the identification approach or algorithms they applied (see last column of Tables 1 and 2).

Hansell et al. (2003) [37] performed a study to examine the validity of routine data sources on COPD and asthma in the United Kingdom (UK). The authors used national data from different sources to obtain information about general practitioner contacts, symptoms, mortality, and emergency hospital admissions. The General Practice Research Database, which is a commercially available database of information on general practice diseases and prescriptions in UK, yielded information about inhalers prescribed in primary care and about earlier or current COPD diagnosis [37].

Wilchesky et al. (2004) [38] performed a study determining sensitivity and specificity of the diagnoses derived from claims data in Canada. Diagnoses were obtained from the medical records of approximately 15,000 patients (used as the “gold standard”) and were compared to the diagnoses in the administrative database of this sample. Sensitivity and specificity were analyzed for the following two methods of COPD identification: (1) recorded diagnosis from the physician claims, and (2) using physician claims diagnostic codes in the year preceding the study [38].

Lacasse et al. (2005) [39] examined the validity of COPD diagnosis in a large administrative dataset from the Quebec health insurance agency (RAMQ, Canada) by comparing it with data from the National Health Survey. RAMQ includes prescription data (drug name and dispensation date) on all prescriptions filled for registered patients ≥65 years of age and for patients with social security. RAMQ also contains information on diagnostic and therapeutic procedures that are performed in hospitals and ambulatory facilities, but does not provide information about spirometry, medication during hospitalization or nursing home stays, and home oxygen use. Outpatients as well as inpatients were considered in this study. All entries matching the diagnosis of COPD, using ICD-9 codes 490–492 and 496, were obtained [39].

Mapel et al. (2006) [11] developed an identification algorithm for the undiagnosed COPD patients using administrative claims data of Lovelace Health Plan, a health maintenance organization serving New Mexico, USA. Patients with new COPD diagnosis during the study period were matched by sex and age to as many as three control subjects. In order to identify preclinical COPD, authors captured all outpatient encounters, hospitalizations, and outpatient pharmacy prescription fills with a time period of 2 years prior to COPD diagnosis. COPD patients were recognized if they were aged ≥40 years with one or more records of COPD diagnosis (ICD-9 codes: 491, 492, and 496) listed on discharge. In the study population of about 41,500 patients, the developed algorithm had 60.5% sensitivity and 82.1% specificity. The reference standard for this analysis was a COPD diagnosis extracted from medical records, based on ICD codes [11].

In 2010, Mapel et al. [42] performed another study to determine if outpatient pharmacy claims can be used for identification of COPD patients (≥40 years, one or more outpatient or inpatient claims, ICD-9 codes: 491–492, 496). To identify drugs that were related to COPD in the years before the diagnosis, a conditional logistic regression model was built with COPD status as the dependent variable and sex, age, and medication use as independent variables. In order to validate the algorithm, it was used in two other databases. The final algorithm identified patients with a specificity of 70.5% and a sensitivity of 60.6%. The reference standard was at least one inpatient or at least two outpatient claims with a COPD diagnosis in the medical records, based on ICD codes [42].

Mapel et al. (2011) [44] performed a cross-sectional administrative claims data analysis to study a new methodology of COPD identification in a large managed care database in the USA. The information was obtained from a dataset of 19 health plans across the USA, about 7.8 million cases. COPD patients were recognized if they fulfilled one of the following three criteria: (1) 40 years or older, plus one emergency room visit or one hospitalization with COPD (491, 492, 496) listed as a discharge diagnosis; or (2) 40 years or older, plus two COPD professional claims with different dates of service; or (3) 40 years or older, plus a COPD-related surgical procedure (e.g., lung volume reduction) [44].

Akazawa et al. (2008) [40] assessed the economic burden of undiagnosed COPD by comparing costs and healthcare utilization in a sample of matched controls (*N* = 81,322) and newly diagnosed COPD patients (*N* = 28,968) in the 1 year period preceding the initial diagnosis. United Healthcare provided pharmacy and medical claims data for this study. COPD was identified using the following three criteria: (1) hospital or emergency department claim with a COPD diagnosis code: 491–492, 496; (2) physician claims with a COPD diagnosis, with another claim having the same code but a different date of service; or (3) physician claims containing a COPD ICD-code and drug-based algorithms [40].

Heins-Nesvold et al. (2008) [41] evaluated the similarity of documented healthcare utilization with patient-reported use, wants and needs in the US. For this reason, two data sources were utilized: (1) managed care administrative database, which includes medical and pharmacy claims data of 7782 cases, and (2) a survey mailed to 1911 Minnesota COPD patients. Patients were identified as ≥40 years old, continuous enrolment during study period, at least one claim with a diagnosis of COPD (ICD-9 codes: 491–492, 496) [41].

Cooke et al. (2011) [24] developed a predictive model using administrative data to identify COPD patients. Data was obtained from the US Department of Veterans Affairs, including outpatient and inpatient databases, pharmacy records, demographic data, and primary ICD-9 codes (491–492, 493.2, and 496), providing a study population of about 9600 individuals. COPD was defined as (1) FEV1/FVC ratio less than 0.70 (indicates COPD) and (2) FEV1/FVC ratio at the lower limits of normal. In total, 4564 had an FEV1/FVC < 0.70. The best model additionally included ≥6 albuterol (a short-acting beta agonist) metered dose inhalers, ≥3 ipratropium (an anticholinergic) metered dose inhalers, ≥1 outpatient ICD-9 code, ≥1 inpatient ICD-9 code, and age. This model reached a sensitivity of 72% and a specificity of 74%, compared to spirometry as a gold standard [24].

Following their analysis published in 2011, in 2012 Dalal et al. [45] assessed in a cohort of 1936 patients whether initiation of a fixed dose combination therapy (fluticasone propionate/salmeterol combination (FSC)), compared to continued or new anticholinergic (AC) therapy, has an impact on the subsequent exacerbations occurrence following an initial exacerbation. Data were obtained from a US healthcare database, the Ingenix Impact National Benchmark database, which includes demographic data, inpatient, outpatient, laboratory results and pharmacy claims. A claim with IDC-9 codes of 491–492 and 496 was considered to represent a diagnosis of COPD [45].

Austin et al. (2012) [26] performed a study using five administrative health databases from Canada, linked using an encrypted insurance number. The Ontario Chronic Obstructive Pulmonary Disease database contains data on people with COPD diagnosis, identified by physician billing claims or hospital discharges with following ICD-9 codes: 491, 492, or 496, or ICD-10 codes: J41, J42, J43, or J44. In a case verification study, with expert opinion as the reference standard (Gershon et al. 2009), the algorithm had a sensitivity of 85.0% and a specificity of 78.4%. A COPD case was only considered an incident case of COPD when the individual patient did not have any COPD claims during the last 5 years [26].

Make et al. (2012) [46] documented and evaluated medication use patterns for COPD patients. Based on guidelines, medication use and adherence, as well as care indicators were analyzed. Data was obtained from the PharMetrics database, which contains 19 health plans across the United States. COPD patients were identified if they were 40 years or older and fulfilled any of the following criteria: (1) an emergency room visit or hospitalization with ICD-9: 491–492, 496; or (2) two professional COPD claims with different service dates; or (3) a COPD-related surgical procedure [46].

Gini et al. (2013) [47] performed a study to estimate the prevalence of COPD, ischemic heart disease, heart failure and diabetes mellitus (DM). They compared the derived estimates with the Italian National General Practitioners’ Medical Record Database and national health survey prevalence estimates. Analyzed data based on the VALORE project was obtained from four sources: (1) hospital discharge records using ICD-9 codes, (2) drug dispensing records using ATC codes (Anatomical, Therapeutic, Chemical Classification System codes) for drug classification, (3) disease-specific exemption from co-payment using ICD-9 codes, and (4) Inhabitant Registry, providing demographic information (sex, year of birth) and identifier of the doctor in charge. The analyses show that for COPD patients the estimates from administrative data were within the confidence intervals of the survey estimates in four regions [47].

Macaulay et al. (2013) [48] studied a COPD severity prediction model, with the Geisinger Health System (GHS) data. Claims data captured resource use (hospital, medical and pharmacy claims) both in and outside of GHS. Electronic health records included present and predicted values of spirometry. Patients with COPD ICD-9 code (491, 492, or 496) and electronic health record spirometry results were selected. Using the Global Initiative for Chronic Obstructive Lung Disease (GOLD) guidelines and spirometry, patients were classified into three groups (severe/very severe, mild/moderate and GOLD-unclassified). In order to categorize COPD severity, a regression model was developed using data from 3 months before and after the last spirometry. COPD severity was predicted for 62.7% of patients with a sensitivity of 50.0, 52.2, and 77.5%, and a specificity of 90.5, 80.0 and 70.4%, for severe/very severe, mild/moderate and GOLD-unclassified, respectively. The reference standard was COPD diagnosis (using ICD-9 codes) and electronic health record results from at least one spirometry test [48].

Yawn et al. (2013) [49] performed a study to establish associations between the use of inhaled corticosteroids (ICS) in patients with a new COPD diagnosis and a dose-related increase in the risk of pneumonia. They used US claims databases, and examined drug prescriptions and medical claims from two MarketScan® databases (Commercial Claims and Encounters, Centers for Medicare and Medicaid Services Supplemental and Coordination of Benefits, with information on clinical utilization, expenditures, and enrolment in inpatient or outpatient services). Included patients had a diagnosis of COPD (ICD-9491, 492, and 496). The study sample consisted of 135,445 patients. Identification of patients was based on COPD-related emergency department visits or admissions, or at least two office visits related to COPD [49].

Dore et al. (2014) [50] performed a study among initiators of a LABA to evaluate the accuracy of claims data for classifying COPD and prevalent asthma. The Normative Health Information Database was used (UnitedHealth Care, USA). ICD-9 codes (491.2, 492.8, and 496) were observed. The National Drug Codes were used for drug identification. All cases had COPD or asthma ICD-9 code on claims in the period from the 6 months prior to the index date. A random sample of medical records was used to verify the diagnoses from each of the four following categories of patients (in total, 370 patients): (1) one or more claims for asthma – ICD-9493, (2) at least one claim for COPD – ICD-9: 491.2, 492.8, 496, (3) claims for both COPD and asthma, (4) without a claim for COPD or asthma. Having at least one COPD claim in the 6 months before the index date resulted in a positive predicted value (PPV) of about 82%, among recipients of inhaled anticholinergic drugs, men and older patients, the PPV was more than 90% [50].

Erdem (2014) [51] analyzed the prevalence of chronic illnesses within the Medicare fee-for-service users in the USA. Data were used from the Chronic Conditions Public Use Files (PUFs). Administrative data for all Medicare fee-for-service users can be found in PUFs. Among all available data in the PUFs, COPD is also included. Algorithms that search for a certain ICD-9 code, Current Procedural Terminology, or the Healthcare Common Procedure Coding System in the beneficiary’s Medicare fee-for-service claims was used as the indicator [51].

Aldrich et al. (2015) [53] aimed to estimate COPD prevalence and potential misreporting using published algorithms for COPD patient identification among low-income adults in the USA, aged 40 to 79 years. The Medicare and Medicaid Services database was used. COPD was identified under the following circumstances: one or more hospitalizations or emergency department visits with an ICD-9 code 491, 492, 496, or at least two visits with different service dates or, alternatively, ICD-9 code 491.21 as discharge diagnosis. Any mentioned COPD diagnosis was explored in order to evaluate the validity of the COPD labelling based on a reference standard of COPD diagnosis in medical records. The sensitivity was 62% and the positive predictive value was 80% for CMS-identified COPD [53].

Crighton et al. (2015) [30] analyzed the epidemiology of COPD and associated health service use in Canada [30]. Four databases were used: (1) The Registered Persons Database, (2) The Canadian Institute of Health Information Discharge Abstract Database, (3) The Ontario Health Insurance Plan Physician Claims database, and (4) the National Ambulatory Care Reporting System databases. Patients included were ≥ 35 years. COPD was identified by: (1) one or more hospitalization related to COPD, and/or (2) one ambulatory claim with ICD-9 code 491, 492, 496 or ICD-10 code J41, J42, J43, J44. This case definition had a 85.0% sensitivity and 78.4% specificity when using physicians’ clinical evaluation as reference standard [30].

Laforest et al. (2016) [55] investigated the frequency and effect of specific comorbidities on all-cause mortality in COPD patients. The Permanent Sample of Health Insurance Beneficiaries, a random sample of the French National Claims Data beneficiaries (SNIIRAM) with linkage between ambulatory and hospital care, was used to select the cohort. COPD patients were identified as (1) ≥45 years of age, with (2) a COPD-related hospitalization (ICD-10 codes J41, J42, J44 and J96.1, while the J96.0 code was accepted only in the presence of J43 or J44), (3) presence of a long-term disease status for COPD (patient suffering from severe chronic conditions), and (4) bronchodilator drugs [55].

Price et al. (2016) [56] examined the comparative effectiveness of albuterol inhalers with and without integrated dose counter for patients with asthma or COPD using US claims data (Clinformatics TM Data Mart database). This database contains medical claims on both primary and secondary health care, laboratory test results, and pharmacy claims. Patients from four up to 64 years of age, having at least one consultation, ED visit, prescription for albuterol, or inpatient admission with COPD diagnosis, were included [56].

Romanelli et al. (2016) [32] estimated the prevalence of COPD using administrative databases. The authors used the city’s hospital discharge register and the cause-specific mortality register as data sources; clinical characteristics were obtained from hospital or outpatient medical records. COPD patients were identified as 40 years or older, with a primary or secondary COPD diagnosis at hospital discharge (ICD-9: 490, 491, 492, 494, 496), or with a COPD diagnosis in hospital or outpatient medical record, or with a FEV1/FVC less than 0.70, or finally COPD as a cause of death. The positive predictive value for COPD in the hospital discharge register was 80.2%, for clinical diagnoses in inpatient medical charts 82.4%, outpatient 81.8, and 90.9% in the cause-specific mortality register. Spirometry had a positive predictive value for COPD of 88% [32].

Lee et al. (2017) [34] performed a study to determine if the COPD patients could be accurately identified using the data available in Electronic Medical Record. Authors used data from the Electronic Medical Record Administrative data Linked Database (EMRALD®) in Ontario. Several COPD algorithms were investigated, as well as their predictive values. An algorithm using the documentation in the cumulative patient profile had a PPV of 95%, and detected 56% of COPD patients. When COPD billing codes (491, 492 or 496) and medication prescriptions (tiotropium, ipratropium, salbutamol or combinations) were included in the algorithm, PPV was 98% with a 52% sensitivity. Algorithms using a combination of more elements from Electronic Medical Record led to a higher sensitivity than when used separately, and a higher PPV, specificity and NPV. The final algorithm resulted in the 77% sensitivity and 96% PPV, and included COPD documentation in the cumulative patient profile, drug prescriptions and COPD billing codes [34].

McGuire et al. (2017) [35] evaluated the risk of incident COPD in rheumatoid arthritis using administrative health data from the Ministry of Health of British Columbia administrative databases on provincially funded health services. This set of data included all physician visits, investigations, and procedures from the Medical Service Plan, as well as hospital data. Furthermore, information on medications use is collected using PharmaNet data, and using vital statistics data on deaths and causes of death. The COPD population was identified based on ICD codes (Revision 9: 491, 492, 493.2, 496 and revision 10: J43 or J44) in hospital and/or outpatient physician visit data (including billing code for COPD) [35].

Westney et al. (2017) [36] investigated the status of comorbidities among Medicaid patients with COPD. The study cohort is obtained from Medicaid Analytic eXtract (MAX) file, originating from Centers for Medicare and Medicaid Services. COPD patients were identified as 18 to 64 years of age, with ICD-9 codes (491.0, 491.1, 491.2, 491.8, 492.xx, 493.2, 494.xx, 496.xx) and one or more inpatient billing claims from the inpatient file or at least two outpatient billing claims [36].

Turner et al. (2018) [58] analyzed the prevalence, features and subtypes of asthma, COPD and asthma COPD overlap. The authors used (1) the HealthCore Integrated Research Database, a health insurance repository of administrative claims data, and (2) patients medical records. Patients were included if they were 40 years of age or older, having two or more COPD diagnoses (ICD-9 codes 491, 492, 496), two or more COPD-related procedures, three or more Generic Product Identifier (COPD medication prescription fills) and two or more Current Procedural Terminology codes for spirometry. Through patients’ medical record review COPD was confirmed by persistent airflow obstruction FEV1/FVC < 0.70 at baseline [58].

## Discussion

This systematic assessment of studies using routine data for the identification of COPD patients includes 38 studies published from January 2000 until October 2018. Until 2010, nine studies were published (on average, a little more than one study per year), while in the next 8 years, an additional 29 studies were published, three times more than the period before 2010. This indicates that use of routine data in COPD patient’s identification is rising. On the other hand, there is a clear discrepancy in where the studies are reporting from: 34 studies present the situation in North America, while only four report on COPD identification practices in Europe (one from United Kingdom and two from Italy and one from France). There were no identified studies in other regions. It is rather unlikely that the identification of COPD implies problems to North America and European countries only. Therefore, there seems to be a compelling need for further research in order to understand how other countries cope with this challenge.

In this review, ICD-9 or ICD-10 coding was the most frequently used instrument to identify COPD patients, adopted in 90% of studies. Hospitalization and age data were provided for the target population in the majority of the studies, followed by ambulatory data, physician claims, and drug prescription data. It was not surprising that only five studies used spirometry findings and only one study used data regarding home oxygen use, as this information is usually not contained in claims databases. Combinations of these identification criteria were used in order to identify COPD patients in routine data (as shown in Tables 1 and 2).

Four studies used other methods than ICD coding: Gershon et al. (2009) and Gershon et al. (2013) used age limitation (older than 35) as an indicator, in addition to one or more claims for hospitalization or ambulatory care for COPD. Dalal et al. (2011) and Raymakers et al. (2017) used age restriction (patients older than 40 years, and 50 years respectively) and pharmacotherapy claims. Offering alternative identification approaches, these studies are of paramount interest for our research.

It is noteworthy that the algorithm described and previously validated by Gershon et al. (2009) has been used in 13 out of 38 studies. Gershon, in six of her studies, uses an algorithm defined by ≥35 years, one COPD hospitalization and/or one ambulatory claim (sensitivity 85% and specificity 78.4%) [23, 25, 27–29, 33]. Austin et al. (2012), Crighton et al. (2015), Westney et al. (2017), Doucet et al. (2016) and McGuire et al. (2017) uses the same algorithm, while Vozoris et al. (2014) and Vozoris et al. (2015) takes in both publications (different population) into account Gershon’s highly specific COPD definition (sensitivity of 57,5% and specificity of 95,4%) which includes three or more ambulatory claims in a 2 year period, and one or more hospitalizations for COPD [26, 30, 31, 52, 54].

The premise of our study is that identification algorithms identified through these studies would be useful for countries with limited evidence from routine/administrative data, in general and in particular for countries where ambulatory ICD codes are not available. Austria is a notable example of this situation, struggling to achieve the best possible information with alternative approaches.

An Austrian attempt to derive ICD codes from routine data was performed in the project “ATC to ICD: Determination of the reliability for predicting the ICD code from the ATC code”, published by Weisser et al. [59], who tried to deduce the ICD code using ATC code (Anatomical, Therapeutic, Chemical Classification System, which is used for pharmaceutical products) from routine outpatient data, an area of the Austrian health care system where ICD codes are missing. In this project the authors showed what would be the most feasible way to assign ICD codes to an ATC code, with use of data available in the Main Association of Austrian Social Insurance Institutions. Additional information used for the analysis was available in this database: sex, year of birth, medication dose, prescription date and medication issue date.

Summarizing our findings, the most elaborate approach to identify COPD patients using routinely available records uses pharmacotherapy data (LABA, SAAC, LAAC, theophylline and inhaled corticosteroids). Particularly for the outpatient sector, in the fields of administrative/social insurance data, pharmacotherapy data is the most reliable and certainly the richest source of information available, if the ICD code is unavailable.

### Limitations

Our review has several limitations. Publication bias may occur because the studies focusing on this specific identification problem may be of interest only in a very limited context (e.g., national interest, health insurance). Our literature search was restricted to Medline via PubMed and Google Scholar. Additionally, a hand search of included studies, only in the English and German languages, was conducted. In the identified published papers, the basic data was frequently not available to review.

The general dilemma of the kind of studies we reviewed is that identification algorithms often lack a gold standard. While Cooke et al. (2011) [24] use spirometry as a gold standard, Romanelli et al. (2016) [32] report spirometry to have a PPV for COPD of (only) 88%. Other authors rely on expert opinion, but there is no common knowledge regarding the estimation of inter-observer variability. Due to the lack of a specific risk of bias tool, we used the method of algorithm validation and the resulting sensitivity within our studies to judge the risk of bias. Although the choice of any threshold should be explicitly informed by a rational decision criterion or an explicit false positive/false negative trade-off, this was missing in most of the studies. However, for the comparability within our review, it was positive that most studies, which applied a validated algorithm, had thresholds leading to a sensitivity of around 80%.

Regarding the generalizability of evidence, the majority of studies are reporting on patients from the USA or Canada. Due to possible diverse identification approaches worldwide, different health systems or datasets, the algorithms reported by some authors in this review might not be applicable to other regions. Based on the fact that different datasets were used, also the identification criteria were diverse between the studies. This could induce the imperative to create many diverse algorithms and, at the same time, makes it difficult to form one unique algorithm that could be applicable to any health care system.

## Conclusion

A variety of different criteria have been used to identify COPD. In general, it can be concluded that the more criteria are combined, the more accurate is the detection of COPD patients in terms of sensitivity and specificity. Drug data is by far the most comprehensive source of information if used alone. The most promising criteria set in data environments where ambulatory diagnosis codes are lacking is the inclusion of other illness-related data with special attention to pharmacotherapy data, and to ATC code if available. In order to obtain more substantial insights on reliable detection of COPD patients from routine datasets, further research should focus on the application of internal and/or external validation approaches.

## Data Availability

All data and material are available in published, mentioned and referenced studies.

## Abbreviations

AC: Anticholinergic
ATC: Anatomical, Therapeutic, Chemical Classification System
CMS: Centers for Medicare and Medicaid Services
COPD: Chronic obstructive pulmonary disease
CPT-4: Current Procedural Terminology
Db: Database
DM: Diabetes mellitus
ED: Emergency department
EHR: Electronic health record
EXE: Disease specific exemptions database
FEV1: Forced expiratory volume in 1 second
FEV1/FVC: The proportion of the forced vital capacity exhaled in the first second
FSC: Fluticasone propionate/salmeterol combination
FVC: Forced vital capacity
GHP: Geisinger Health Plan
HCPCS code: Healthcare Common Procedure Coding System
HSV: Hauptverband der österreichischen Sozialversicherungsträger (Main Association of Austrian Social Insurance Institutions)
ICD: International Classification of Diseases
ICD-10: International Classification of Diseases X Revision
ICD-9: International Classification of Diseases IX Revision
ICS: Inhaled corticosteroids
LAAC: Long-acting anticholinergic bronchodilators
LABA: Long-acting beta2-agonist bronchodilators
LAMA: Long acting muscarinic antagonists
MDI: Metered dose inhalers
MOXXI: The Medical Office of the 21st century
NA: Not available
NPV: negative predictive value
PPV: positive predictive value
PUFs: Public Use Files
RAMQ: Quebec health insurance agency
SAAC: Short-acting anticholinergic bronchodilators
SABA: Short acting beta agonist
SAMA: Short acting muscarinic antagonists
SP: Study population
